# Neuronal diversity in the caudate nucleus: A comparative study between camel and human brains

**DOI:** 10.1002/ar.25555

**Published:** 2024-08-08

**Authors:** Juman M. Almasaad, Ziad M. Bataineh, Sami Zaqout

**Affiliations:** ^1^ Department of Basic Medical Sciences, College of Medicine King Saud Bin Abdul Aziz University for Health Sciences (KSAU‐HS) Jeddah Saudi Arabia; ^2^ King Abdullah International Medical Research Centre (KIAMRC) King Abdulaziz Medical City Jeddah Saudi Arabia; ^3^ Department of Anatomy, Faculty of Medicine Jordan University of Science & Technology Irbid Jordan; ^4^ Department of Basic Medical Sciences, College of Medicine, QU Health Qatar University Doha Qatar

**Keywords:** camel, caudate nucleus, comparative anatomy, Golgi, neuronal diversity

## Abstract

Caudate nucleus (CN) neurons in camels and humans were examined using modified Golgi impregnation methods. Neurons were classified based on soma morphology, dendritic characteristics, and spine distribution. Three primary neuron types were identified in both species: rich‐spiny (Type I), sparsely‐spiny (Type II), and aspiny (Type III), each comprising subtypes with specific features. Comparative analysis revealed significant differences in soma size, dendritic morphology, and spine distribution between camels and humans. The study contributes to our understanding of structural diversity in CN neurons and provides insights into evolutionary neural adaptations.

## 1 BACKGROUND

The caudate nucleus (CN) is a significant subcortical structure composed of gray matter, pivotal to the overall structure and function of the brain. Its ontogenic development coincides with the curvature of the telencephalic vesicle, leading to its characteristic C‐shaped morphology (Yelnik, 2002). Consisting of the head, body, and tail, the CN stretches into the anterior horn along the floor of the lateral ventricle, curving downward and backward into the temporal lobes within the basal ganglia (Lee et al., 1995; Yin & Knowlton, 2006). Functionally, the CN plays a crucial role in controlling voluntary movements and regulating emotions (Reiner et al., 1998).

Together with the putamen (PuN), the CN constitutes the striatum (St), displaying a striated appearance attributed to bands of gray matter connecting the CN's head to the PuN via the anterior limb of the internal capsule (Lee et al., 1995). The striatum, integral in functions such as reward‐based learning, habit formation, and action selection (Cardinal, 2006; Everitt & Robbins, 2005; Hollerman et al., 2000; Rolls, 1994), dynamically interacts with diverse cortical and subcortical regions (Frank & Claus, 2006; Haber et al., 2006; Morgane et al., 2005; Robbins et al., 1989), with dysfunction implicated in clinical and psychiatric conditions (Grace, 1991; Grant et al., 2006; Heinz et al., 1994; Hyman et al., 2006; Ikemoto, 2004; Jentsch et al., 2000; Volkow et al., 2004). The CN, forming a neural circuit linking different regions of the cerebrum (Yin & Knowlton, 2006), governs voluntary movements, and disturbances in its connections can affect both movement and emotional regulation, contributing to various clinical disorders (Cicchetti et al., 2000; Graybiel et al., 1994).

Discrepancies in cytological studies of adult rat neostriatal cell types identify medium‐sized (types I‐IV), giant (type V), and small (type VI) cells, each exhibiting distinct characteristics, adding complexity (Dimova et al., 1980). Additional rat studies classify neurons based on size, dendritic branching, spines, and axonal characteristics, suggesting diverse functional roles (Mensah & Deadwyler, 1974). Comparative Golgi studies in shrews and voles reveal three main neuron types contributing to neostriatum diversity (Wasilewska et al., 2007). Monkey studies unveil at least six neuronal types in the striatum, emphasizing the complexity of neural organization (DiFiglia et al., 1976). In the human striatum, five neuron types and four axon types highlight intricate architecture (Graveland et al., 1985). Electron microscopy studies in cats reveal essential characteristics of CN fine structure, including small neurons without Nissl bodies and myelinated fibers in the neuropil, indicating a functional organization primarily involving axodendritic synapses (Kemp & Powell, 1971).

Human CN development involves three consecutive parts forming the head, originating from medial, lateral, and intermediate striatal elevations, with variations observed in different mammalian species (Hewitt, 1958, 1959). Golgi studies elucidate the diversity of CN neurons, categorized into projective or efferent neurons and local or interneurons, showing a phylogenetic increase in types and subtypes (Eder et al., 1980).

The CN in camels (*Camelus bactrianus*) has been described as a sizable gray mass forming the floor of the lateral ventricle, with the PuN externally covering the pallidum (Zhaohui Xie et al., 2011). Despite the wealth of research exploring various aspects of neuronal structures in diverse animal brains, investigations specifically focusing on the neuronal architecture of the CN in camels remain notably sparse. Our study aimed to address this gap by delving into the specific neuronal characteristics of the CN, given its distinct functional and pathological significance. Particularly implicated in associative learning and the control of voluntary movement, the CN served as the focal point of our investigation. By delineating morphological characteristics and conducting comparative analyses with humans and other species, our study contributes to a growing body of research covering neuronal characteristics in various regions of the camel brain (Al‐Hussain et al., 2021, 2022; El‐Dwairi et al., 2023; Zaqout et al., 2012). Importantly, our findings deepen our understanding of neural diversity and the functional implications across species.

## 2 MATERIALS AND METHODS

We used modified Golgi techniques to investigate diverse neuron types within the CN of two mammalian species: camels and humans.

### 2.1 Experimental design

1. An analysis was conducted to identify distinct neuronal types within the CN of both camels and humans.
2. The description focused on morphological features of various neuronal types identified within the CN of both camel and human brains.
3. A comparative analysis was performed between the neuronal characteristics observed in the CN of camels and those found in humans, aiming to delineate similarities and differences between the two species.

### 2.2 Animals and human samples

The methodology of this study involved examining specimens of the CN from both human and camel subjects. Postmortem CN samples were obtained from a total of 8 individuals (3 men and 5 women) aged between 32 and 66 years, who had passed away in road accidents. These samples were sourced from King Abdullah University Hospital, Irbid, with ethical approvals obtained from the Jordan University of Science and Technology Institutional Review Board (IRB). Camel CN specimens were obtained from five male camel brains, ranging in age from 2 to 3 years (juveniles). These brains were sourced from slaughtered animals and purchased from local butcher shops. Notably, no animals were sacrificed explicitly for the purpose of this study, leading to the waiver of approval from the Institutional Animal Care and Use Committee (IACUC). Following collection, the camel brains were preserved within 0.5–2 h postmortem in distilled water on ice. They were then transported in sealed, labeled boxes to the laboratory. Both human and camel CN specimens were immediately fixed by immersion in 10% formaldehyde for a duration exceeding 1 month to ensure comprehensive preparation for subsequent analysis.

### 2.3 Modified Golgi stain method

Two Golgi methods were used for optimal impregnation (Graveland et al., 1985).

#### 2.3.1 Rapid Golgi procedure

1. A solution was prepared by dissolving 0.2 gm osmium tetroxide and 2.5 gm potassium dichromate in 100 mL deionized water (DiFiglia & Carey, 1986).
2. Caudate blocks (5 mm thick), fixed in 10% formalin for over a month, were cut, dried, and immersed in the prepared solution for 10 days.
3. The blocks were subsequently dried without washing and immersed in a 0.75% silver nitrate solution at 37°C for a duration of 5 days.

#### 2.3.2 Golgi‐Kopsch method (double impregnation)

1. A mixture was prepared by dissolving 5 g of potassium dichromate in 99 mL of deionized water and adding 1 mL of formaldehyde (Graveland et al., 1985).
2. Caudate blocks, each 5 mm thick and fixed for over a month in 10% formalin, were cut, dried, and immersed in the mixture for 4 days.
3. Following a sequential treatment process, the blocks were immersed in 3.5% potassium dichromate for 4 days, followed by a 2‐day treatment in 0.75% silver nitrate. Subsequently, they underwent another 4‐day immersion in 3.5% potassium dichromate, concluding with a 3‐day treatment in 0.75% silver nitrate.

Blocks impregnated via both procedures underwent further processing:

4 Embedding in paraffin wax, cutting into 100–120 μm sections on a rolling digital microtome.
5 Dehydration in absolute alcohol for 2 h, cleaning in xylene for 5 min, and mounting on slides under cover slips.

### 2.4 Photomicrographs

We investigated intriguing and well‐impregnated neurons using a Nikon light microscope equipped with a photography system. The neurons underwent comprehensive examination, measurement, and photography to document their intricate morphological characteristics. All images were processed using Adobe Photoshop Version 25.6.0.

### 2.5 Quantitative parameters

1. The mean diameters of somata were measured from well‐impregnated neurons.
2. The numbers of primary dendrites and their branching order were determined for well‐impregnated neurons.

### 2.6 Neuronal morphological classification within the caudate nucleus

The primary criteria for classification encompassed:

1. Presence, types, density, and distribution of dendritic spines.
2. Somal size.
3. Number and branching order of dendrites.
4. Varicosities.

### 2.7 Limitations of the study

The perfusion process is challenging to apply for large mammals such as camels. Additionally, the Golgi method is highly selective, and the degree of completeness of impregnation can vary. Our study focused on the overall features of the neurons regardless of gender and age differences. However, it would be interesting to conduct further research with a larger number of samples from different age and gender groups to investigate any potential differences. Future studies with controlled perfusion processes from the earliest possible stages could provide improved data.

## 3 RESULTS

### 3.1 Quality of Golgi impregnations

The Golgi staining method, staining a very low percentage (1%–5%) of neurons, facilitated the detailed study of individual cells (Kemp & Powell, 1971; Zaqout & Kaindl, 2016). Successful staining was achieved across all blocks, yet the effectiveness and fidelity of cellular staining differed among the techniques used. The Golgi‐Kopsch method primarily highlighted densely spiny neurons, whereas the rapid Golgi method showcased a wider array of cell types.

### 3.2 Neuronal classification in the caudate nucleus

Based on our classification criteria, the CN of each species revealed the presence of three primary types of neurons:

#### 3.2.1 Rich‐spiny neurons (type I)

**FIGURE 1:**
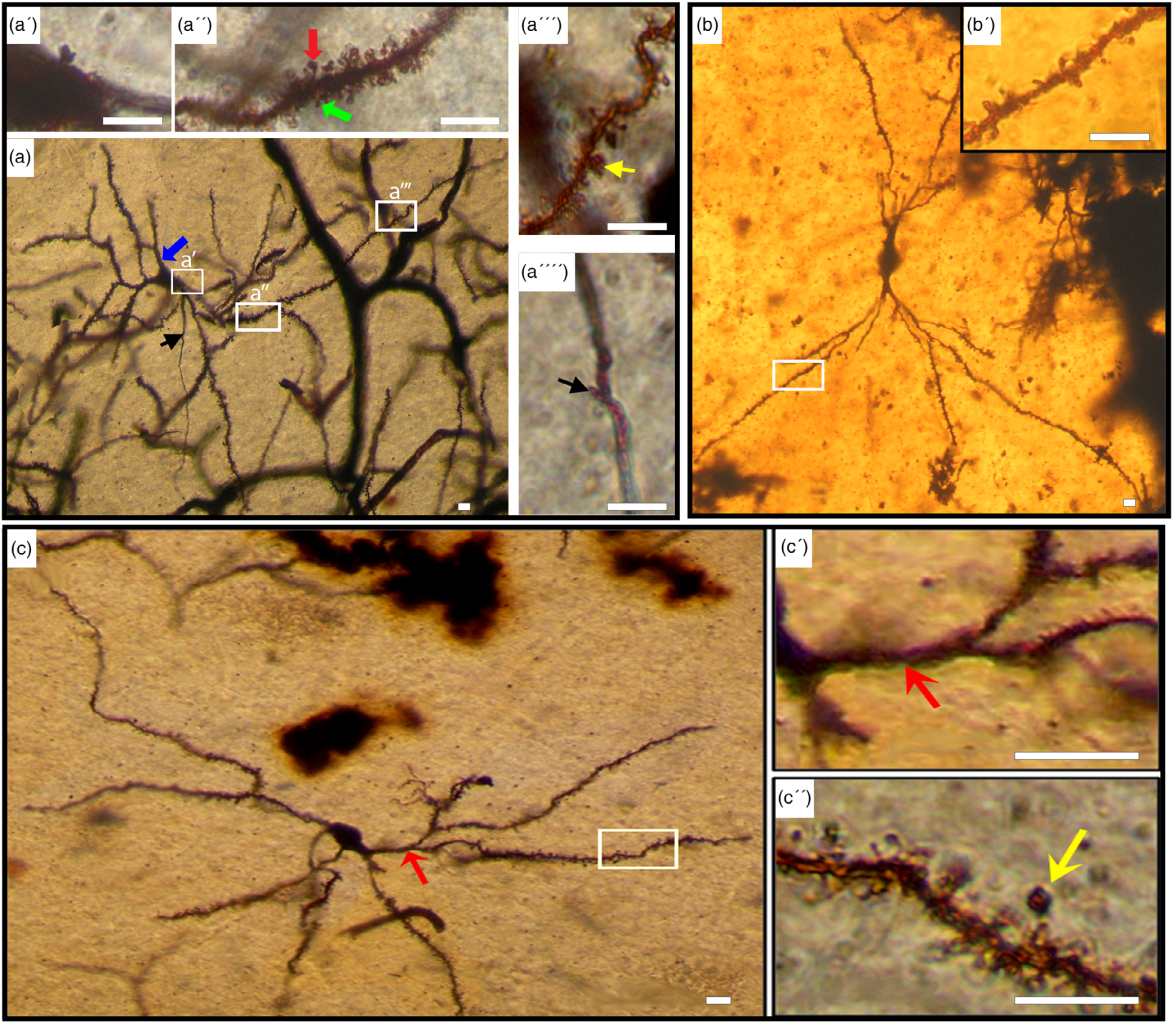
Rich spiny medium‐sized neurons (Type I, Subtype A). (a) Composite photomicrograph illustrating a well‐impregnated type I neuron of medium size in the camel caudate nucleus (CN). The inset (a′) highlights a protrusion on the cell body. Primary dendrites lack spines (blue arrow), whereas the proximal segment of secondary dendrites (inset a″) displays a dense covering of spines. Spine density gradually decreases along the distal branches (inset a‴). Various types of spines are present, including hair (green arrow, inset a″), mushroom‐shaped (red arrow, inset a″), and stubby (yellow arrow, inset a‴). Occasionally, protrusions can be observed on the axon, as indicated by black arrows in (a) and the inset (a⁗). (b) Photomontage showing rich spiny neurons (Type I) of medium size in the human CN. Two thick dendritic trunks emerge from the oval soma. Various types of spines are depicted in the inset (b′). (c) Photomontage of a relatively low spine density neuron (Type I) of medium size in the camel CN. The cell body is oval, and primary dendrites are smooth (red arrow, inset c′). Inset (c″) reveals dendrites with various types of spines, and the yellow arrow indicates an appendage with a long stem. Scale bars, 10 μm; DIC images.

**FIGURE 2:**
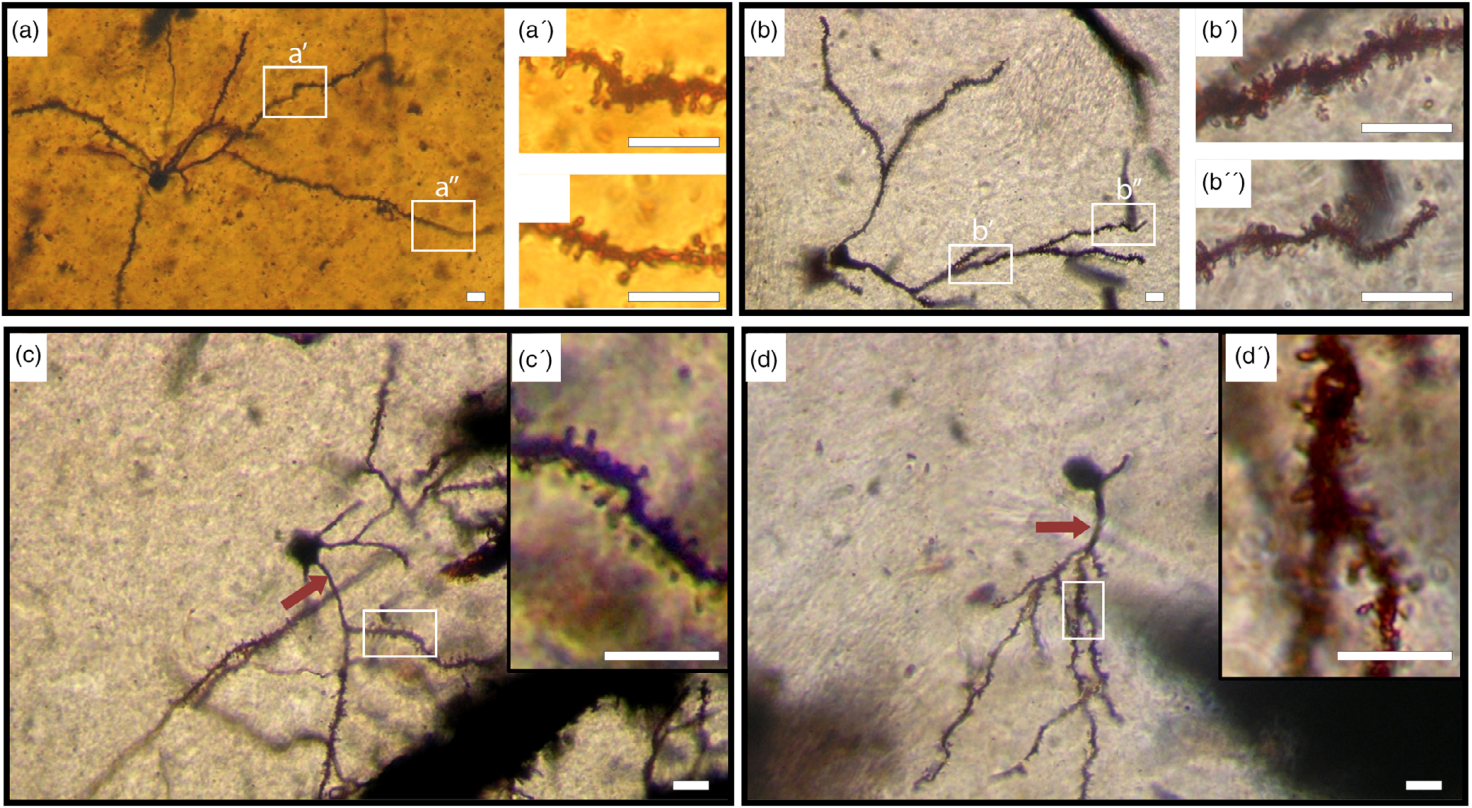
Rich spiny small‐sized neurons (Type I, Subtype B). (a) Photomontage illustrating a rich spiny neuron (Type I) of small size in the camel caudate nucleus (CN). The cell body is triangular, giving rise to two long primary dendrites that bifurcate up to fourth‐order branching. Spines are visible along all dendritic segments. Insets (a′) and (a″) depict the distribution of dendritic spines on proximal and distal dendrites, respectively. (b) Photomontage showing a rich spiny neuron (Type I) of small size in the human CN. The soma is rounded, with four primary dendrites. Insets (b′) and (b″) highlight the proximal and distal dendrites, respectively, showing a notable decrease in spine density toward the terminal segments. (c) Photomicrograph of a relatively low spine density neuron (Type I) of small size in the camel CN. Long, smooth primary dendrites are noted (red arrow). Inset (c′) demonstrates the spine density in the area indicated by the rectangle in figure (c). (d) Photomicrograph of a relatively low spine density neuron (Type I) of small size in the human CN. Long, smooth primary dendrites are observed (red arrow). Inset (d′) illustrates the spine density in the area indicated by the rectangle in figure (d). Scale bars, 10 μm; DIC images.

Within this group, two subtypes were observed,

Medium‐Sized Neurons (Subtype A): In both species, the examined neurons (Figure 1a,b) exhibited similar morphology, constituting the fundamental cellular populations in the CN. The cell bodies exhibited a range of 10–18 μm with a mean diameter of 15 μm ± 1.47 (*n* = 80) in camels and 12–22 μm with a mean diameter of 17.5 μm ± 1.75 (*n* = 80) in humans. Various shapes were observed for the somata, including polygonal, rounded, oval, and triangular. Occasional spines were detected on both the soma and primary dendrites. In humans, cells displayed 4–8 smooth dendritic trunks, most dividing up to six times. Conversely, camels exhibited cells with 3–5 smooth dendritic trunks, with division up to four times. Across both species, dendrites extended outward to form either a round‐ or oval‐shaped dendritic field. Dendrites were adorned with various spine types, including hair‐like, stubby, or mushroom shapes (Figure 1a″, a‴). The overall spine density was 20 ± 1.8/10 μm in camels and 8.3 ± 0.7/10 μm in humans. Hair‐like spines were more abundant than other types (73% in humans and 55% in camels, Table 1). Spines were denser in the proximal portion of the dendrites and decreased toward the distal branches (Figure 1a″, a‴). The camel exhibited a higher density of dendritic spines than humans. The axon predominantly originated from the initial segment of the dendritic trunk, with branching axonal collaterals noted within the dendritic domain. Occasionally, protrusions can be observed on the axon, as indicated by the black arrow in (Figure 1a) and the inset (Figure 1a⁗).
  Neurons with a relatively low spine density (Figure 1c) were observed in both mammals. This cell group was characterized by an oval cell body, smaller than neurons with a higher spine density. Cell bodies averaged from 12 to 18 μm with a mean diameter of 14.2 μm ± 1.37 in camels and 15.5 μm ± 1.87 in humans. This group exhibited fewer primary dendrites (two to four) and less branching (two to four orders).
Small‐Sized Neurons (Subtype B): The cell bodies of this group were characterized by measurements of less than 10 μm with a mean diameter of 7.8 μm ± 0.86 (*n* = 20) in camels (Figure 2a) and less than 12 μm with a mean diameter of 9.2 μm ± 1.1 (*n* = 20) in humans (Figure 2b). These somata typically displayed rounded or oval shapes, featuring 2–4 primary dendrites bifurcating up to three orders. In camels, primary dendrites exhibited elongation and sparse spining, whereas in humans, they remained devoid of spines. All types of spines were found in this neuronal type, with the highest density observed on the proximal portion of the dendrites. Notably, camels displayed a higher abundance of dendritic spines compared with humans (Table 1). The axon originated from the cell body, with evident axonal collaterals extending within the dendritic field. Additionally, neurons exhibiting a relatively lower spine density were also observed within this group (Figure 2c,d).

**TABLE 1:** A comparison of rich spiny neurons (type I), sparsely‐spiny neurons (type II), and aspiny neurons (type III) in the camel and human caudate nucleus.

Parameter	Camel	Human
Rich spiny (type I)		
Mean cell body diameter (μm)	15 ± 1.47 (Medium; *n* = 6)	17.5 ± 1.75 (Medium; *n* = 8)
	7.8 ± 0.86 (Small; *n* = 6)	9.2 ± 1.1 (Small; *n* = 6)
Number of primary dendrites	3–5	4–8
Orders of branching	Up to 4	Up to 6
Mean spine density (/10 μm)	20 ± 1.8	8.3 ± 0.7
Hairy spines (%)	55	73
Stubby spines (%)	20	15
Mushroom spines (%)	25	12
Sparsely spiny (type II)		
Mean cell body diameter (μm)	24.2 ± 3.9 (Large; *n* = 5)	26.8 ± 3 (Large; *n* = 4)
	15.2 ± 1.9 (Medium; *n* = 3)	17 ± 2.2 (Medium; *n* = 6)
Number of primary dendrites	2–4	2–6
Mean spine density (/10 μm)	6 ± 1.5	2 ± 0.9
Hairy spines (%)	50	60
Stubby spines (%)	40	35
Mushroom spines (%)	10	5
Aspiny (type III)		
Mean cell body diameter (μm)	28.4 ± 4.3 (Large; *n* = 10)	33.6 ± 5.4 (Large; *n* = 8)
	14.2 ± 2.5 (Medium; *n* = 6)	16 ± 2.9 (Medium; *n* = 4)
	7.5 ± 0.5 (Small; *n* = 5)	9.2 ± 0.6 (Small; *n* = 5)
Number of primary dendrites	1–5	2–11
Varicosities	More	Less

#### 3.2.2 Sparsely‐spiny neurons (type II)

**FIGURE 3:**
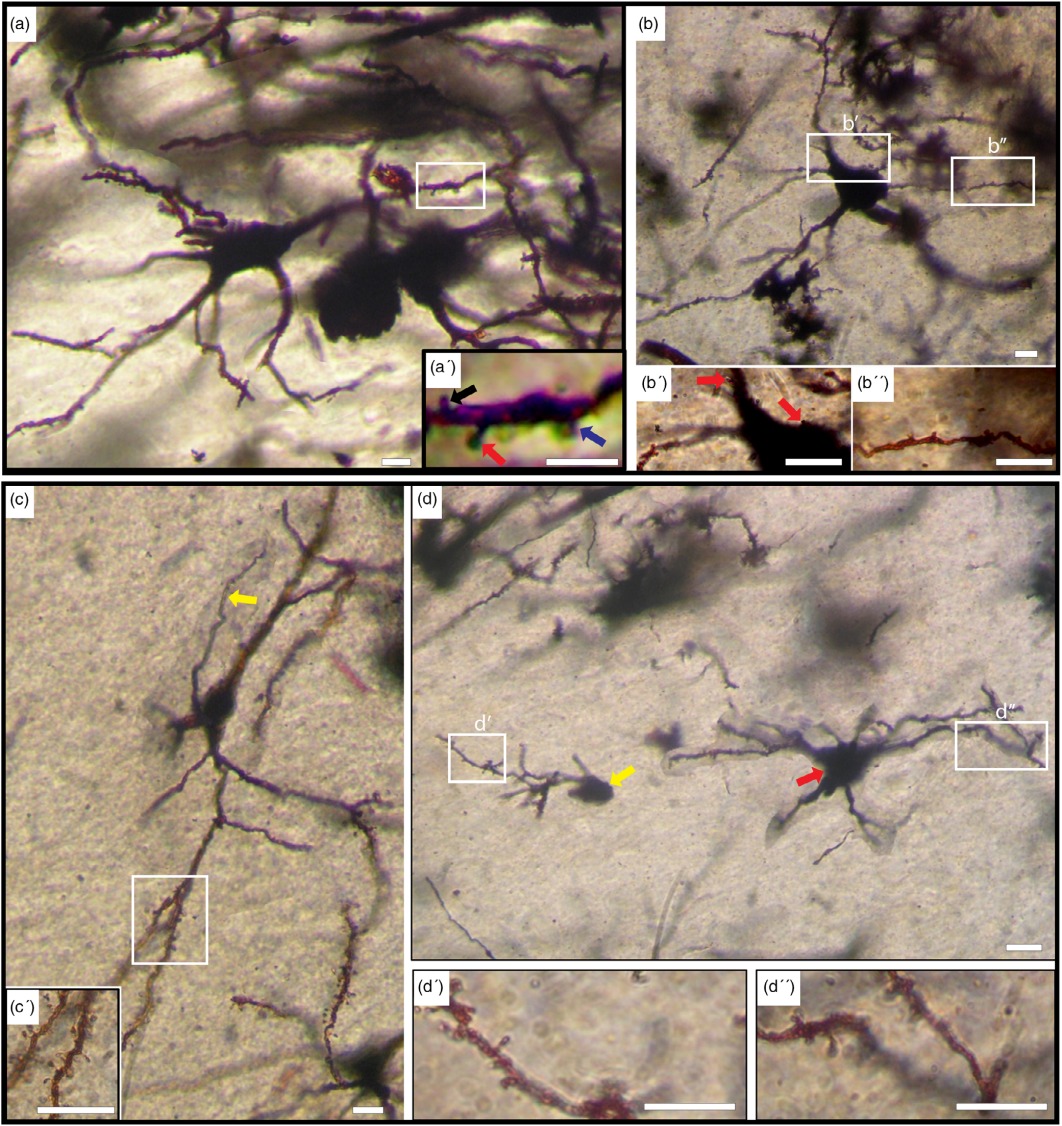
Sparsely spiny neurons (Type II). (a) Photomontage of a sparsely spiny large‐sized neuron in the camel (Type II, Subtype A). Five thick primary dendrites emerge from the soma, sparsely branched with few varicosities, very few spines, and exhibiting a tortuous course. Inset (a′) displays hair‐like (black arrow), stubby‐like (blue arrow), and mushroom‐like (red arrow) spines. (b) Large‐sized Type II neuron in the human. Spines are present on the surface of the cell body and thick primary dendrites (red arrows) as shown in inset (b′). Inset (b″) illustrates dendrites with sparse spines. (c) Photomontage of a medium‐sized sparsely spiny neuron (Type II, Subtype B) in the camel caudate nucleus (CN). The axon is indicated by the yellow arrow. Inset (c′) displays dendrites with a low density of spines. (d) Medium‐sized (red arrow) and small‐sized (yellow arrow) sparsely spiny neurons (Type II, Subtypes B and C respectively) in the human CN. Insets (d′) and (d″) depict dendrites with sparse spines. Scale bars, 10 μm; DIC images.

Only a limited number of cells belonging to this type were successfully impregnated. Based on somal size, this type is further classified into three subtypes:

Large Neurons (Subtype A): The cellular morphology is characterized by a rounded, oval, or triangular shape, with the cell body displaying a diameter surpassing 20 μm and exhibiting a mean diameter of 24.17 μm ± 3.86 (*n* = 10) in camels (Figure 3a). In humans, it measures more than 23 μm with a mean diameter of 26.8 μm ± 3 (*n* = 10) (Figure 3b).
Medium Neurons (Subtype B): The morphology of the cell bodies (highlighted by red arrows in Figure 3c,d) exhibits variation, ranging from rounded to oval or triangular shapes. In camels, the diameter averages from 12 to 18 μm, with a mean diameter of 15.2 μm ± 1.85 (*n* = 10), whereas in humans, it ranges from 12 to 22 μm, with a mean diameter of 17 μm ± 2.2 (*n* = 10).
Small Neurons (Subtype C): Observed only in the human CN (Figure 3 (yellow arrow)), these neurons have rounded cell bodies with a diameter measuring less than 12 μm and a mean diameter of 8.5 μm ± 1.35 (*n* = 10).

In all mentioned variations, somata displayed the presence of spines. Camels exhibited two to four primary dendrites emanating from the cell body, whereas humans displayed two to six primary dendrites. Dendrites were relatively thick, tortuous, and sparsely branched. Remarkably extensive dendritic branching was observed in both medium and large‐sized neurons. Spines of all types (including hairy, stubby, and mushroom‐shaped) were present on all dendritic segments. Detailed differences between camel and human sparsely spiny neurons are outlined in Table 1.

#### 3.2.3 Aspiny neurons (type III)

**FIGURE 4:**
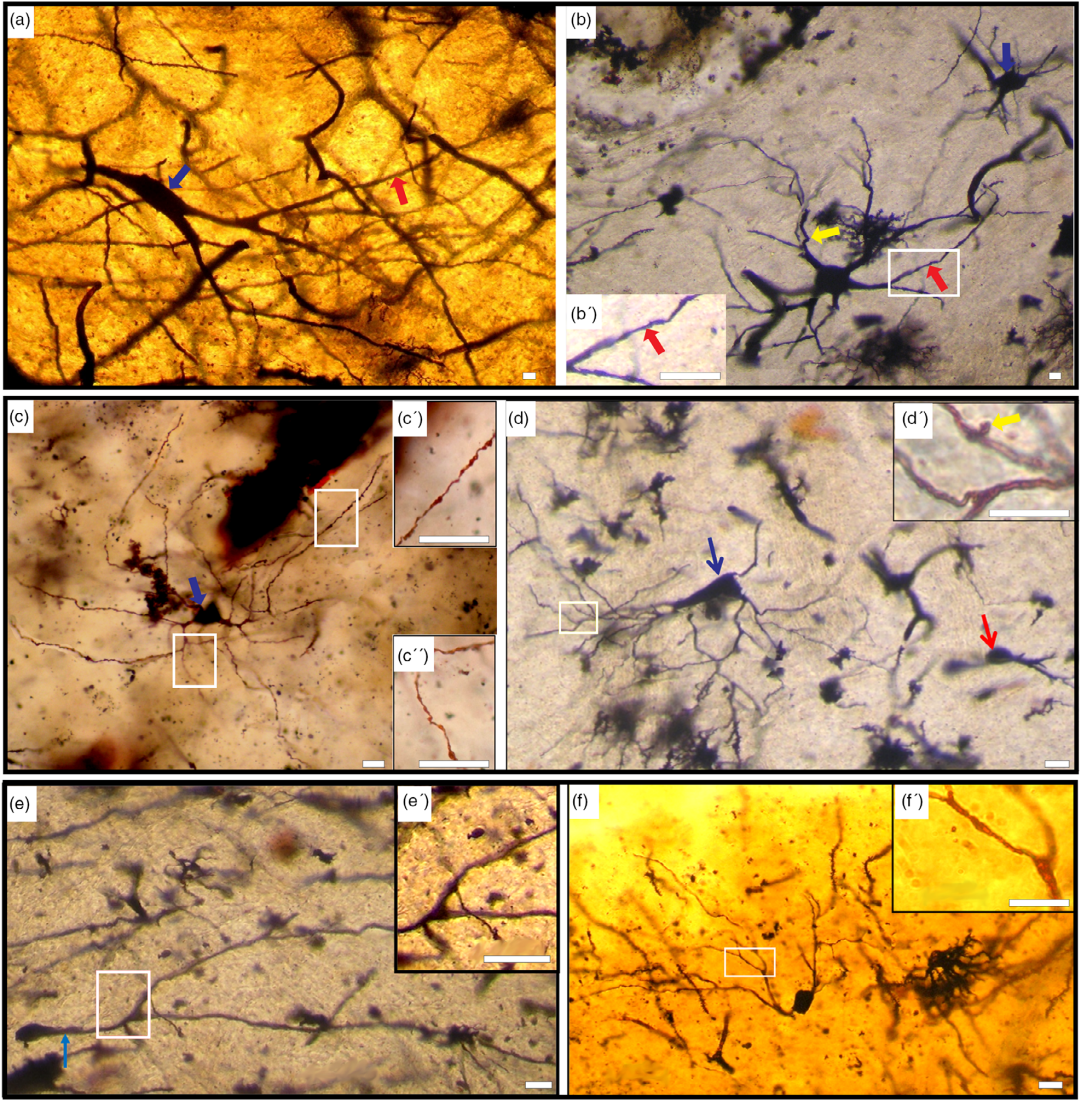
Aspiny neurons (Type III). (a) Photomicrograph of an aspiny large‐sized neuron (Type III, Subtype A; blue arrow) in the camel. The neuron exhibits an oval shape with few dendrites and an absence of dendritic spines (red arrow). (b) Photomontage of an aspiny large‐sized neuron (Type III, Subtype A) in the human caudate nucleus (CN). The cell body is rounded with five primary dendrites, displaying irregular contours with varicosities (red arrow) and a tortuous course (yellow arrow). Inset (b′) shows dendrites without spines. Additionally, a medium‐sized aspiny neuron (Type III, Subtype B; blue arrow) with dendrites having a wavy course is depicted on the top right. (c) Photomontage of a medium‐sized aspiny neuron (Type III, Subtype B; blue arrow) in the camel CN. The cell body is triangular‐shaped with three primary dendrites. Insets (c′) and (c″) demonstrate irregular contours with varicosities in the proximal and distal dendrites, respectively. (d) Photomontage of a medium‐sized aspiny neuron (Type III, Subtype B) in the human CN. The neuron exhibits an oval shape (blue arrow), with thin, smooth contour primary and secondary dendrites. An appendage is present on the surface of the terminal branches (yellow arrow). Additionally, a small‐sized aspiny neuron (Type III, Subtype C; red arrow) with a rounded cell body and one dendritic trunk is observed on the right side of this figure. (e) Photomontage of a small‐sized aspiny neuron (Type III, Subtype C) in the camel CN. The oval cell body has one long, primary dendrite (blue arrow), which bifurcates twice. Inset (e′) highlights the absence of spines in the secondary dendrites and their branches. (f) Photomontage of a small‐sized aspiny neuron (Type III, Subtype C) in the human CN. The oval‐shaped soma gives rise to two primary dendrites that arborize up to four times. Inset (f′) shows the absence of spines in all dendritic segments. Scale bars, 10 μm; DIC images.

This type includes the following subtypes:

Large Aspiny Neurons (Subtype A): These cells (Figure 4a,b) exhibit a lower frequency of impregnation compared with the medium‐sized neurons. In camels, they typically display a triangular or polygonal shape, whereas in humans, they manifest variable shapes, including rounded, oval, triangular, and polygonal forms. The diameter of the somata measures more than 25 μm with a mean diameter of 33.6 μm ± 5.38 (*n* = 5) in humans, and more than 20 μm with a mean diameter of 28.35 μm ± 4.25 (*n* = 5) in camels. The number of primary dendrites ranges from 2 to 5 in camels and 3 to 11 in humans. Notably, dendrites exhibit greater ramification in humans (up to eight orders) compared with camels (up to four orders).
Medium Aspiny Neurons (Subtype B): The somata exhibit either triangular or polygonal shapes, with an average diameter ranging from 10 to 20 μm and a mean diameter of 14.2 μm ± 2.52 (*n* = 10) in camels (Figure 4c). Conversely, in humans (Figure 4b (blue arrow), d), they present rounded, oval, triangular, or polygonal shapes, with a diameter spanning from 12 to 25 μm and a mean diameter of 16 μm ± 2.91 (*n* = 10). Two to four primary dendrites emanate from the cell body in camels, exhibiting up to six‐order branching, whereas in humans, three to six primary dendrites are observed, resulting in up to an eight‐order branching. The distal branches exhibit irregular configurations and numerous varicosities, particularly pronounced in camels when compared with humans. Additionally, terminal branches may display additional appendages.
Small Aspiny Neurons (Subtype C): These cells represent the least abundant subset within the neuronal population (Figure 4d (red arrow), e, f). The somata of the small aspiny neurons measure less than 12 μm in both mammals, exhibiting a mean diameter of 7.5 μm ± 0.47 (*n* = 5) in camels and less than 10 μm with a mean diameter of 9.2 μm ± 0.6 (*n* = 5) in humans. Somata typically assume rounded or oval shapes and are characterized by one to four long dendritic trunks bifurcating up to three times in camels and up to five times in humans. The cell body and primary dendrites have a smooth appearance, while varicosities might be visible on the secondary dendrites and their branches.

Regarding Type III neurons, there are no notable differences between the two species except for variations in cell body size and the number of primary dendrites and their branching, as outlined in Table 1.

## 4 DISCUSSION

The overall morphology of the basal ganglia in both camels and humans, including the CN, closely resembles that described in previous studies (Lee et al., 1995; Zhaohui Xie et al., 2011). The classification of neurons in the CN has been a subject of extensive investigation across various mammalian species, including cats (Kemp & Powell, 1971), rats (Mensah & Deadwyler, 1974), monkeys (DiFiglia et al., 1976), and humans (Graveland et al., 1985). Various studies have used a range of criteria for classification, including factors such as cell body size (Kemp & Powell, 1971), the presence of dendritic spines (DiFiglia et al., 1976; Fox, Andrade, Hillman, & Schwyn, 1971; Fox, Andrade, Schwyn, & Rafols, 1971), and the termination site of axons (Difiglia et al., 1978).

Numerous studies have delineated a multitude of neuronal types within the CN of various species, including rabbits, cats, and humans (Eder et al., 1980). Likewise, investigations into the monkey neostriatum utilizing the Golgi‐Kopsch perfusion method unveiled a minimum of six neuronal types (DiFiglia et al., 1976). Unique classifications have also emerged for other species, such as the European bison, which identified five distinct neuron types (Rowniak et al., 1994), and the guinea pig, which described four types (Szteyn et al., 2000). Conversely, observations in rats solely revealed aspiny neurons within the neostriatum (Takagi et al., 1984).

### 4.1 Neuronal classification in the caudate nucleus of camels and humans

Our study aimed to comprehensively analyze the neuronal characteristics of the CN across its various regions, including the head, body, and tail. However, upon examination, we found no significant differences in neuron types, soma size, or dendritic tree patterns among these regions. This uniformity led us to conclude that treating the CN as a single entity is sufficient for the purposes of our study. In addition, our analysis did not reveal any significant disparities between male and female individuals in the human CN, suggesting no discernible differences across age or sex categories.

In alignment with the established criteria encompassing dendritic spine characteristics, cell body dimensions, dendritic attributes such as number and branching order, and the presence of varicosities, our classification of neurons in the camel and human CN identifies three primary types. These neuron types are defined based on their spiny or aspiny nature and further subdivided for detailed characterization.

The first type, Rich‐spiny neurons (Type I), is distinguished by its subdivision into two subtypes, namely, Medium‐sized (Subtype A) and Small‐sized (Subtype B), with differentiation based on soma size. Rich‐spiny neurons exhibit variations in dendritic spine types, density, and distribution, as well as distinctive cell body features. The morphological differences within this type are crucial for understanding the structural diversity of neurons in the CN.

The second type, Sparsely‐spiny neurons (Type II), encompasses two subtypes—Large‐sized (Subtype A) and Medium‐sized (Subtype B), observed in both camel and human. These neurons are distinguished by a reduced prevalence of dendritic spines and adhere to the predetermined criteria for classification. The recognition of these subtypes contributes to the comprehensive understanding of sparsely‐spiny neuron diversity across species.

The third type, Aspiny neurons (Type III), includes three subtypes—Large‐sized (Subtype A), Medium‐sized (Subtype B), and Small‐sized (Subtype C). This type, defined by the absence of dendritic spines, exhibits variations in soma size, primary dendrite number, and varicosity presence. Notably, a unique subtype (Small‐sized, Subtype C) is exclusive to the human CN, emphasizing species‐specific differences in the aspiny neuron category.

### 4.2 Comparative analysis of neuronal types in the caudate nucleus: Camel versus human

In the comparative analysis of neuronal types within the CN of camels and humans, our investigation revealed notable distinctions that contribute to our understanding of species‐specific neuroanatomical features. The camel's CN neurons exhibited characteristic differences in soma size, dendritic morphology, and spine distribution when compared with their human counterparts.

Neurons within the camel CN presented diminutive somata and comparatively slender, less branched dendritic trunks in contrast to their human counterparts. Remarkably, spiny neurons (Type I and Type II) in camels manifested an elevated presence of spines, along with a distinctly irregular contour of dendrites and varicosities, a contrast starkly evident when compared with humans (Table 1).

Medium‐sized rich spiny neurons (Type I, Subtype A) in camels showed a variable soma shape, with three to five dendritic trunks dividing up to four times, contrasting with the four to eight dendritic trunks branching up to six times in human neurons. Furthermore, spines of different types were more abundant in humans, particularly the hair‐like spines.

Small‐sized rich spiny neurons (Type I, Subtype B) in camels exhibited rounded or oval‐shaped somata, smaller than those in humans. Variations in primary dendrite number, bifurcation pattern, and spine distribution were observed between the two species.

Sparsely‐spiny neurons (Type II) displayed two subtypes in camels (large‐sized and medium‐sized), whereas humans exhibited three subtypes (large‐, medium‐, and small‐sized). Differences in primary dendrite characteristics, branching patterns, and spine density were evident, with camel neurons displaying a higher spine density.

Approximately 5% of striatal cells were identified as aspiny interneurons, housing an array of diverse neurotransmitters (Kawaguchi et al., 1995). In our study, large aspiny neurons (Type III, Subtype A) in camels had triangular or polygonal‐shaped somata, smaller than those in humans. Variations in primary dendrite number, thickness, and ramification were observed.

Medium aspiny neurons (Type III, Subtype B) in camels exhibited rounded, oval, or triangular‐shaped somata, smaller than those in humans. Differences in primary dendrite and branch numbers, along with varicosity visibility, were noted. Small aspiny neurons (Type III, Subtype C) displayed variations in primary dendrite bifurcation and varicosity presence between the two species.

### 4.3 Comparative study of neuronal types in the caudate nucleus: Camel and human in comparison with other species

The neurons identified in this study within the CNi of camels and humans exhibit similarities with those observed in previous studies. In both species, rich spiny medium‐sized neurons (Type I, Subtype A) align with type I neurons in monkeys, characterized by oval shapes and up to seven smooth primary dendrites, extending well beyond the dendritic field with numerous collaterals (DiFiglia et al., 1976). Similar correspondence exists with type I neurons in European bison (Rowniak et al., 1994) and guinea pigs (Szteyn et al., 2000), displaying rounded shapes with five to eight dendritic trunks that bifurcate near the cell bodies (measuring 10.15 μm), giving rise to fine collaterals. Analogous traits are observed in type I neurons in cats, characterized by rounded or polygonal cell bodies housing a plethora of dendritic spines, sometimes presenting challenges in distinguishing them as distinct entities even under high magnification (Kemp & Powell, 1971).

Our findings regarding the types and distribution of spines in rich spiny neurons align with previous observations in humans and monkeys (Graveland et al., 1985). Although our study reveals the highest spine density on secondary dendrites, a different pattern is noted in rats, where most secondary dendrites lack spines, and spine numbers steadily increase, reaching a maximum on tertiary dendrites (Dimova et al., 1980). In rats, medium‐sized projection neurons exhibit axonal collaterals with two distinct branching patterns: one confined to the originating dendritic domain, whereas the other displays thicker, prolonged axonal branching extending beyond the dendritic domain (Kawaguchi et al., 1995). Our investigation aligns with the former pattern, characterized by axon collaterals primarily within the dendritic field. These axonal branches conclude within the neostriatum, aiming at both striatal interneurons and other medium spiny cells, thus playing a substantial part in the intrastriatal production of GABA (Bolam et al., 2000; Kita, 1993; Park et al., 1980). Additionally, another investigation proposes that spiny neurons could serve as the central integrating component in the camel neostriatum (Parent et al., 1995). Medium spiny neurons, serving as major targets for both local and extrinsic afferents, predominantly express GABA as their primary neurotransmitter (Oertel & Mugnaini, 1984; Ribak et al., 1979), alongside other neuroactive peptides (Beckstead & Kersey, 1985; Guzman et al., 1993).

The presence of protrusions on the axons of (Type I, Subtype A) neurons reported in our current study suggests the formation of axo‐axonic synapses. Since action potential generation occurs at the initial axonal segment, these synapses could significantly influence the output of neurons with spiny initial axonal segments (Huang & Rasband, 2018). In a previous study, we observed and reported axonal protrusions in type IIIa neurons in the camel cuneate nucleus (Zaqout et al., 2012). Additionally, electron microscopy studies of the striatum and cortex in cats and rats have shown that axonal spines can be postsynaptic specializations (Kemp & Powell, 1971; Sloper & Powell, 1973; Westrum, 1970).

Although small‐sized rich spiny neurons (Type I, Subtype B) were often overlooked in rats, monkeys, humans, and guinea pigs, they were identified in the bank vole neostriatum (Wasilewska et al., 2007). Described as having rounded multipolar and triangular perikarya, these neurons possess two to six primary dendrites that bifurcate up to six times, corresponding to type III neurons found in rabbits, cats, monkeys, and humans (Eder et al., 1980). These neural entities, alongside efferent elongated axonal small neurons and medium‐sized spiny neurons, comprise the primary cellular contingent within the neostriatum of mammals, representing a substantial proportion, nearly 95%, of the overall striatal cellular population (Kemp & Powell, 1971).

In the monkey neostriatum, a distinct neuronal subtype termed large‐sized sparsely‐spiny neurons (Type II, Subtype A) has been identified, characterized by elongated cell bodies, long thick dendrites, and a comparatively sparse distribution of spines. Although these neurons exhibit shared features with medium‐sized sparsely‐spiny neurons (Type II), such as the presence, distribution, and density of spines, they are classified within this particular subtype (DiFiglia et al., 1976). Corresponding to type V neurons in cats, giant neurons in rats, and one sub‐population of multipolar neurons (Type III) in guinea pigs, these neurons exhibit perikarya measuring 32–40 μm, sending off 7–9 thin, richly arborized dendritic trunks covered with spines (Kemp & Powell, 1971; Mensah & Deadwyler, 1974; Szteyn et al., 2000). Our findings in sparsely‐spiny neurons (Type II) align with those observed in monkeys and guinea pigs (DiFiglia et al., 1976; Szteyn et al., 2000).

Although we observed one type of medium‐sized sparsely‐spiny neurons (Type II, Subtype B) in camels and humans, two types were described in cats, with Type II neurons having slightly larger cell bodies and polygonal or spindle shapes, and Type III neurons being rounded with four to five long slender dendrites (Kemp & Powell, 1971). In rats, two types of neurons have been described: sparsely‐spiny medium‐sized neurons (type II) with four to five highly branched dendrites and sparsely‐spiny medium‐sized neurons (type III) giving off up to seven dendrites thicker than those of medium spiny cells (type I) and markedly varicose (Dimova et al., 1980).

In humans, a unique neuronal subtype termed Small‐sized sparsely‐spiny neurons (Type II, Subtype C) has been identified, characterized by rounded perikarya and the emergence of two or three smooth primary dendrites that branch up to three times. Notably, these neurons are exclusively observed in humans and are not found in camels. Characterized by evenly distributed hairy and mushroom‐like spines, the findings align with a study of human neostriatum (Graveland et al., 1985). This type of neuron is not commonly observed in previous studies in cats, rats, common shrews, bank voles, guinea pigs, and monkeys (Kemp & Powell, 1971; Mensah & Deadwyler, 1974; Szteyn et al., 2000; Wasilewska et al., 2007). The functional significance of Type II neurons within the neostriatum remains shrouded in mystery, notwithstanding the observation of elongated axons in human neurons (Graveland et al., 1985). This observation hints at a potential involvement in a segment of the efferent caudate‐nigral pathway, potentially incorporating substance P (Groves, 1983; Ljungdahl et al., 1978).

Large‐sized aspiny neurons (Type III, Subtype A) observed in our study correspond to giant aspiny neurons in European bison, one of the multipolar sub‐populations in guinea pigs, and aspiny neurons with numerous varicose dendrites in monkeys (DiFiglia & Carey, 1986; Rowniak et al., 1994; Szteyn et al., 2000). Described as very large aspiny type III neurons with polygonal‐shaped cell bodies and local axons (Takagi et al., 1984), they also correspond to type I AchE‐positive neurons (Bolam et al., 1984). Acetylcholine, with a slow and not always followed by an increase in firing, has a muscarinic action in these neurons, different from the action of glutamic acid (Bernardi et al., 1976). After conducting an exhaustive investigation using Golgi‐electron microscopy in primates, researchers revealed a diverse array of axonal types forming synapses with large aspiny neurons. These synaptic connections were dispersed throughout the cell, including its initial axon segment (DiFiglia & Carey, 1986). Large‐sized aspiny neurons, comprising only a small fraction of the neuronal population within the neostriatum, play a significant role in modulating synaptic transmission among spiny projection neurons and interneurons (Groves, 1983; Ljungdahl et al., 1978). The primary function of these neurons is believed to involve the regulation of inputs from dopaminergic afferents and the activity of projection spiny neurons (Calabresi et al., 1997, 2000). Current neuroanatomical evidence suggests that acetylcholine innervation of the neostriatum primarily originates from large aspiny interneurons (Calabresi et al., 2000).

Medium‐sized aspiny neurons (Type III, Subtype B) observed in our study exhibit triangular or polygonal‐shaped soma with 2–4 dendrites branching up to six orders in camels and variable soma shapes with 3–6 dendrites bifurcating up to eight orders in humans. Corresponding medium‐sized aspiny neurons described in cats have rounded cell bodies giving rise to 5–7 dendrites with varicose branches (Kemp & Powell, 1971). Medium‐sized aspiny neurons found in common shrews and bank voles exhibit a diverse array of morphologies, ranging from polygonal and triangular to spindle‐shaped and rounded perikarya. Bank voles typically exhibit 2–5 elongated dendrites, whereas common shrews may feature up to 6 dendrites. The trajectory of their dendrites varies from straight to wavy, with some branching into fourth‐order segments in bank voles. Their dendritic field adopts an ellipsoidal configuration, with primary dendrites appearing either smooth or occasionally bearing slight swellings (Wasilewska et al., 2007). In contrast, medium‐sized aspiny type I neurons in monkeys showcase dendrites with varicosities and a slender axon that branches proximal to the soma (DiFiglia et al., 1976). The medium‐sized aspiny neurons of European bison lack spines on both their perikarya and dendritic processes, although certain cells exhibit convoluted dendrites and dendritic enlargement (Rowniak et al., 1994). Particularly noteworthy, medium‐sized aspiny striatal interneurons are recognized for their role in modulating the activity of medium‐sized spiny neurons in humans (Cicchetti et al., 2000). These interneurons' axons predominantly terminate onto the proximal somatodendritic region of projection neurons, establishing symmetrical synaptic connections (Cicchetti et al., 2000).

The structural attributes of small‐sized aspiny neurons (Type III, Subtype C) bear similarities to type V neurons noted in human studies (Braak & Braak, 1982). However, our examination did not yield precise information on axonal termination points or the synaptic connections formed with other neurons. These small‐sized aspiny neurons also correspond to type VI neurons in cats and small aspiny neurons in bank voles. Type VI neurons in cats exhibit irregular cell bodies and between 6 and 9 dendrites with extensive branching (Kemp & Powell, 1971). Similarly, they resemble small aspiny cells observed in rats, featuring dendrites devoid of spines and exhibiting varicosities, often resembling slender, twig‐like branches (Mensah & Deadwyler, 1974).

The presence of an increased number of interneurons in the CN of certain mammals implies a broader range of neuronal types across phylogenesis (Eder et al., 1980). Our study corroborates this notion, revealing discrepancies in neuronal morphology between human and camel CNi. Specifically, human neurons display larger cell bodies than those in camels. Although human neurons exhibit a higher frequency of dendritic trunk bifurcation, camel neurons possess more abundant dendritic spines. These structural differences may be attributed to the evolutionary positioning of Homo sapiens in the Anthropoidea order, which is relatively younger compared with camels in the Artiodactyla order. Noteworthy research indicates a higher proportion of aspiny neurons in the neostriatum of monkeys (23%) compared with mice and rats (4%–5%), suggesting a more substantial role of these interneurons in the neostriatal organizational framework and function in primates versus rodents (Graveland & DiFiglia, 1985).

Omics technologies, such as single‐cell RNA sequencing, have indeed emerged as powerful techniques for exploring cellular diversity, particularly in brain regions. Single‐cell and single‐nucleus RNA sequencing (scRNA‐seq/snRNA‐seq) have the capacity to greatly expand our understanding of neuronal diversity within the caudate nucleus. These technologies analyze gene expression at the single‐cell level, thereby revealing previously unknown neuronal subtypes (Malaiya et al., 2021). For instance, a recent study analyzed thousands of individual interneuron nuclei from the caudate nucleus and putamen, uncovering new insights into the diversity and abundance of these cell types (Fernandez‐Moya et al., 2023). Similarly, other investigators used single‐nucleus RNA sequencing to identify novel neuronal subtypes and diversity in the human brain (Welch et al., 2019). These studies exemplify how advanced omics approaches have broadened our understanding of cellular heterogeneity in brain regions analogous to those examined in our study.

## 5 CONCLUSION

Our study sheds light on the intricate morphological features of CN neurons in camels and humans. We observe differences, such as larger soma sizes and increased dendritic trunk branching in human neurons compared with camels, suggesting possible evolutionary variations. This supports the idea of an advanced human CN and aligns with the concept of increasing neuronal diversity over evolution. Our findings stress the importance of a comprehensive approach that considers both structure and function in future research on CN neural networks. The classification system we present provides a foundation for understanding structural diversity and encourages further exploration of its functional implications. Recognizing species‐specific neuroanatomical variations is crucial for a nuanced understanding of the CN, offering insights into potential functional implications across species.

## AUTHOR CONTRIBUTIONS

**Juman M. Almasaad:** Investigation; writing – original draft; visualization; formal analysis; data curation; methodology. **Ziad M. Bataineh:** Supervision; resources; funding acquisition; writing – original draft; project administration; conceptualization. **Sami Zaqout:** Conceptualization; methodology; validation; software; writing – review and editing; supervision; data curation.

## FUNDING INFORMATION

This project was funded by Deanship of Research at the Jordan University of Science and Technology (JUST). Open Access funding is supported by Qatar University and Qatar National Library.

## CONFLICT OF INTEREST STATEMENT

The authors declare no conflict of interest.

## Data Availability

The datasets used and/or analyzed during the current study are available from the corresponding author on reasonable request.
